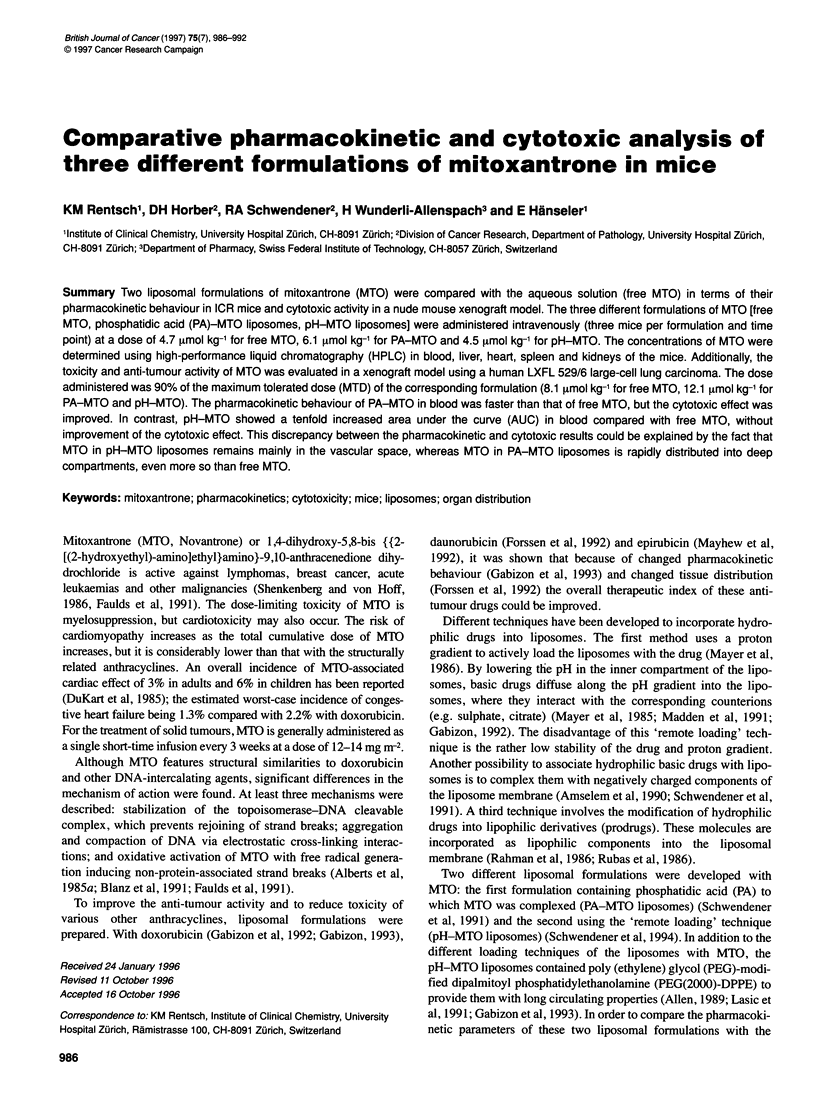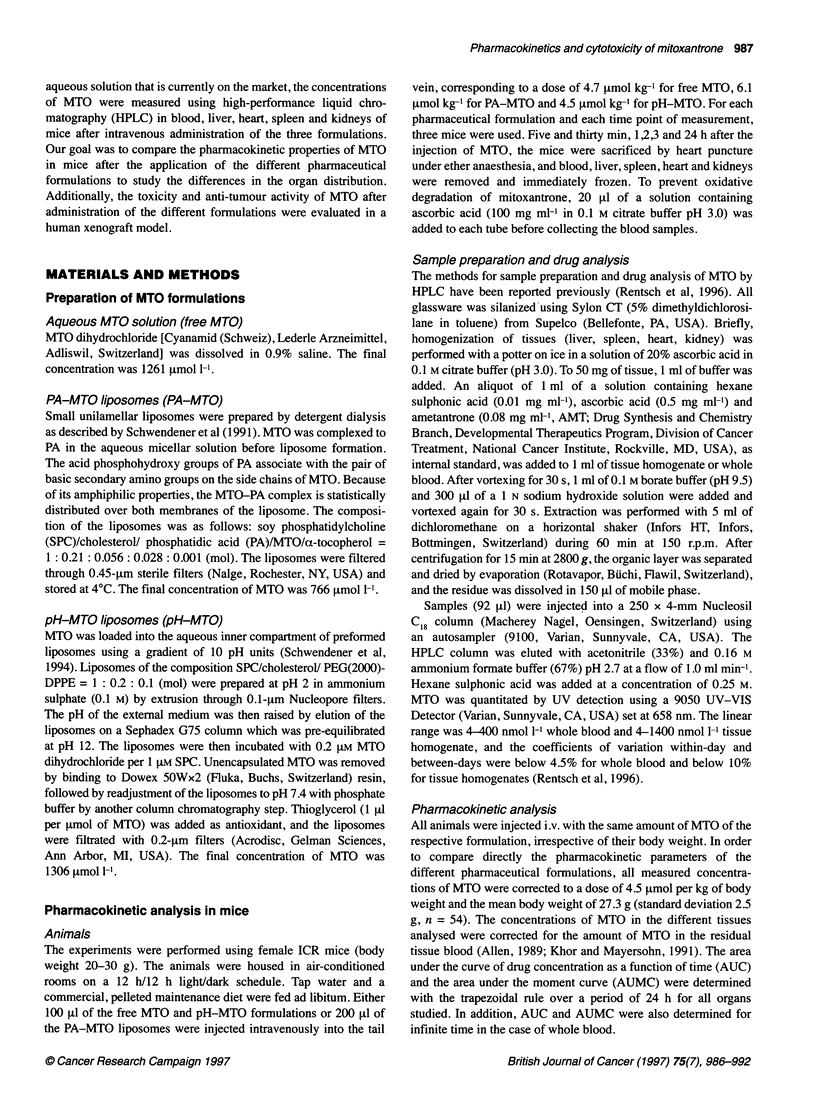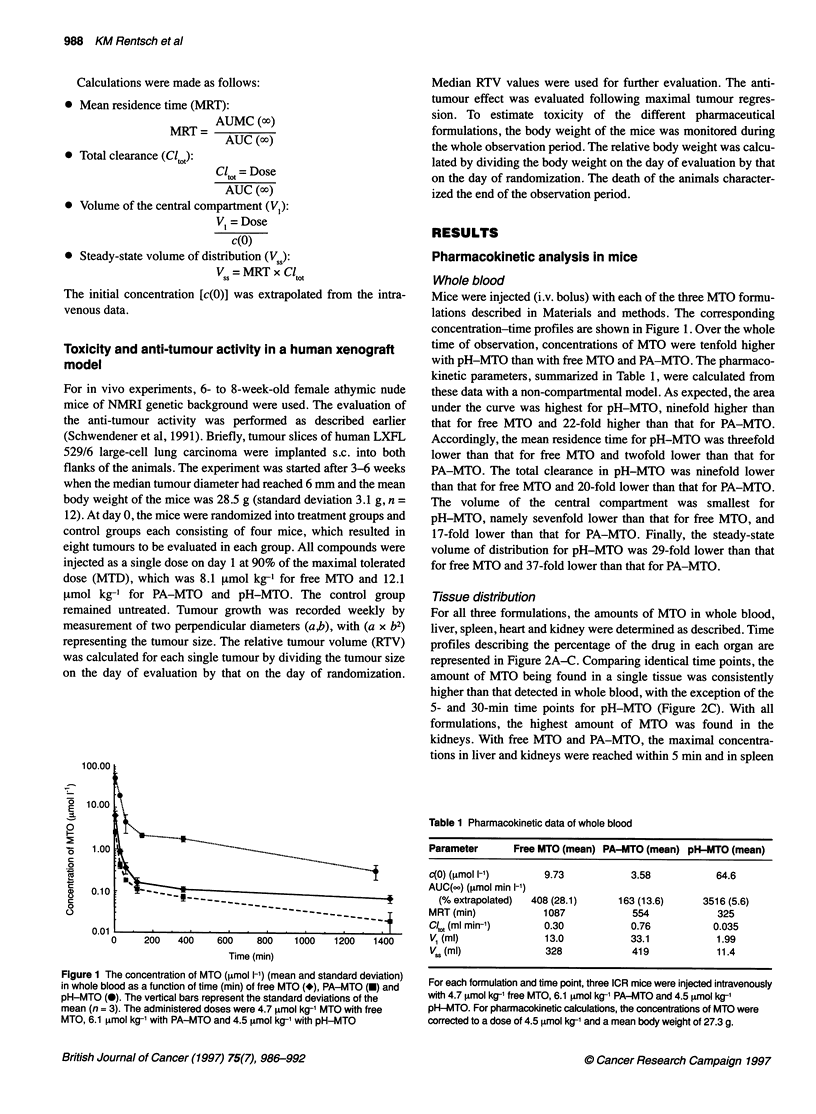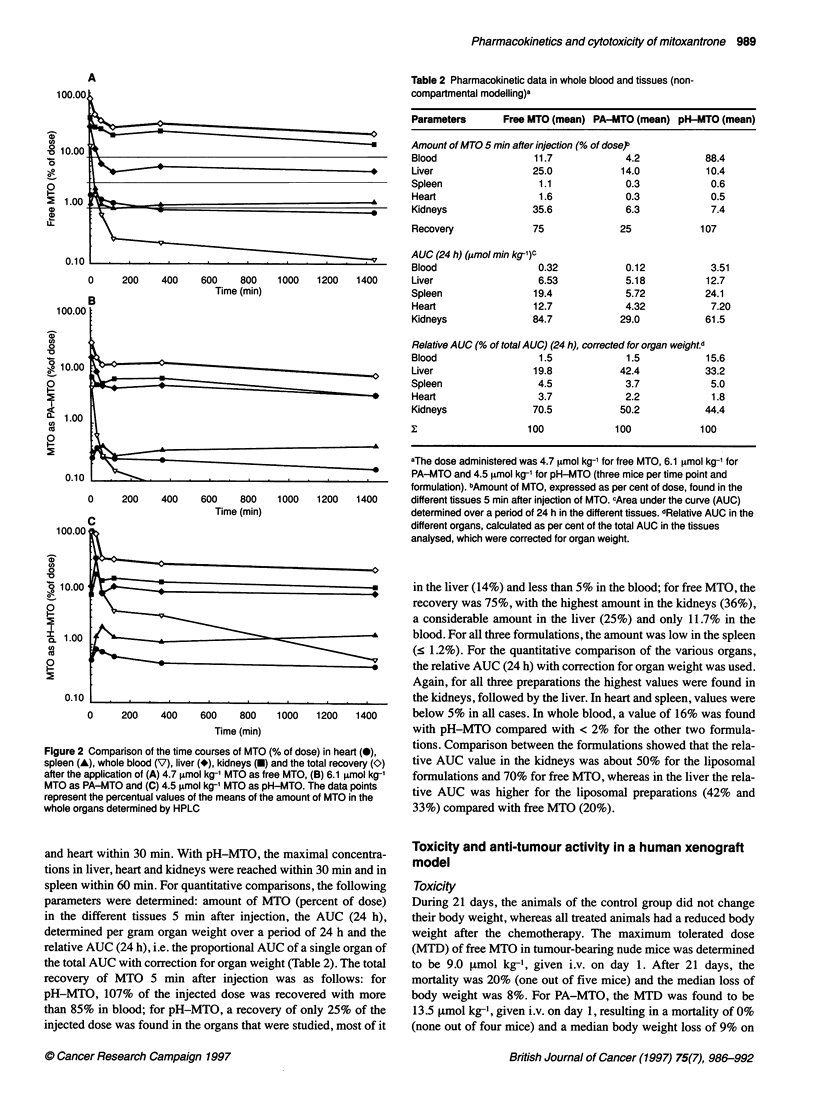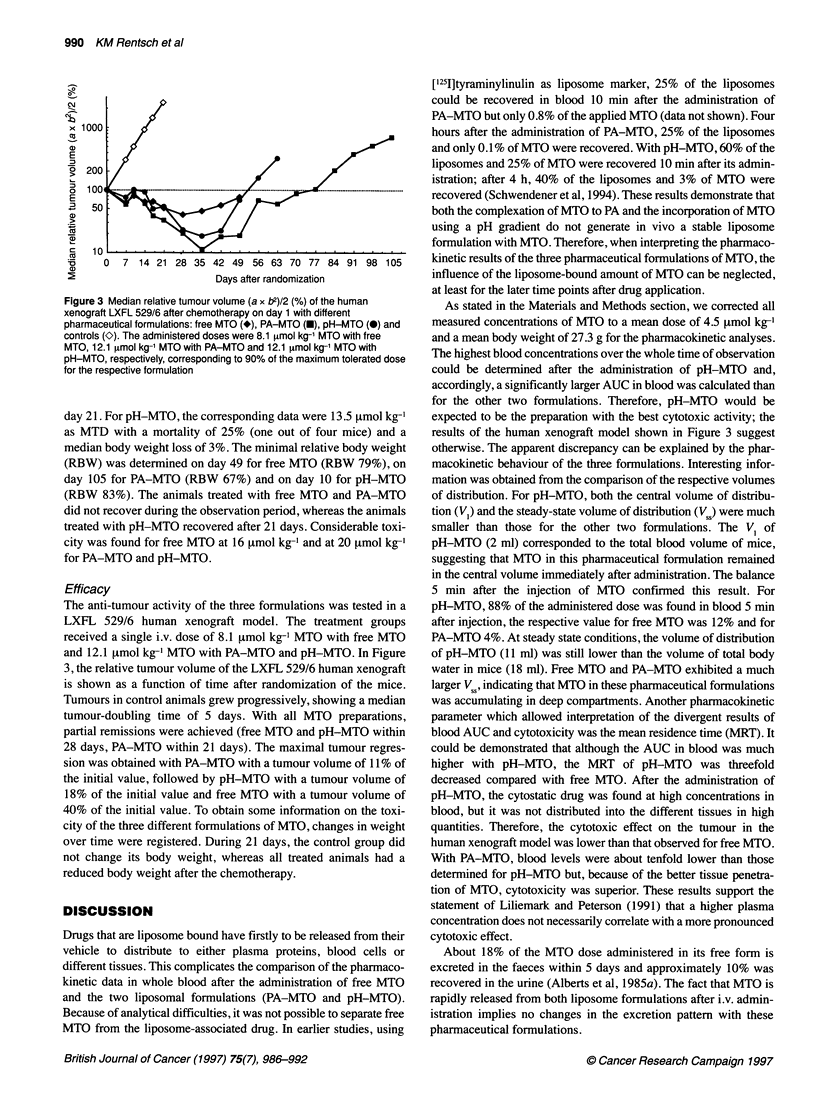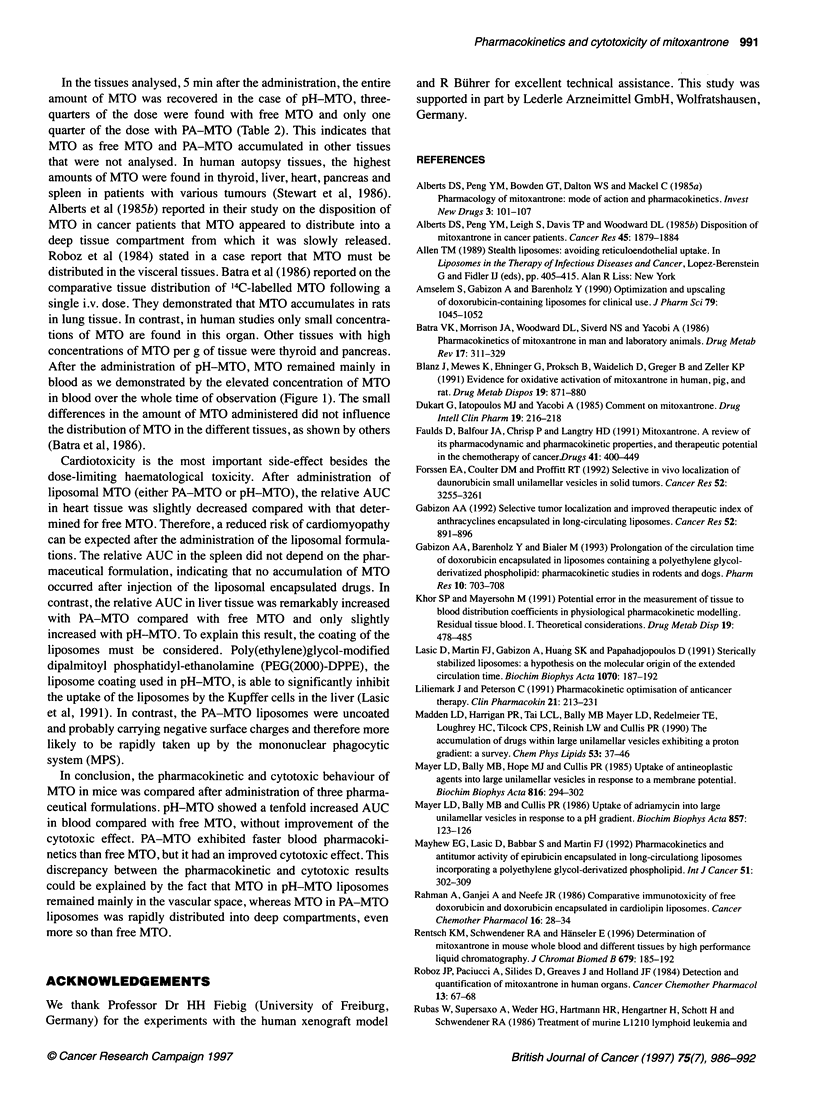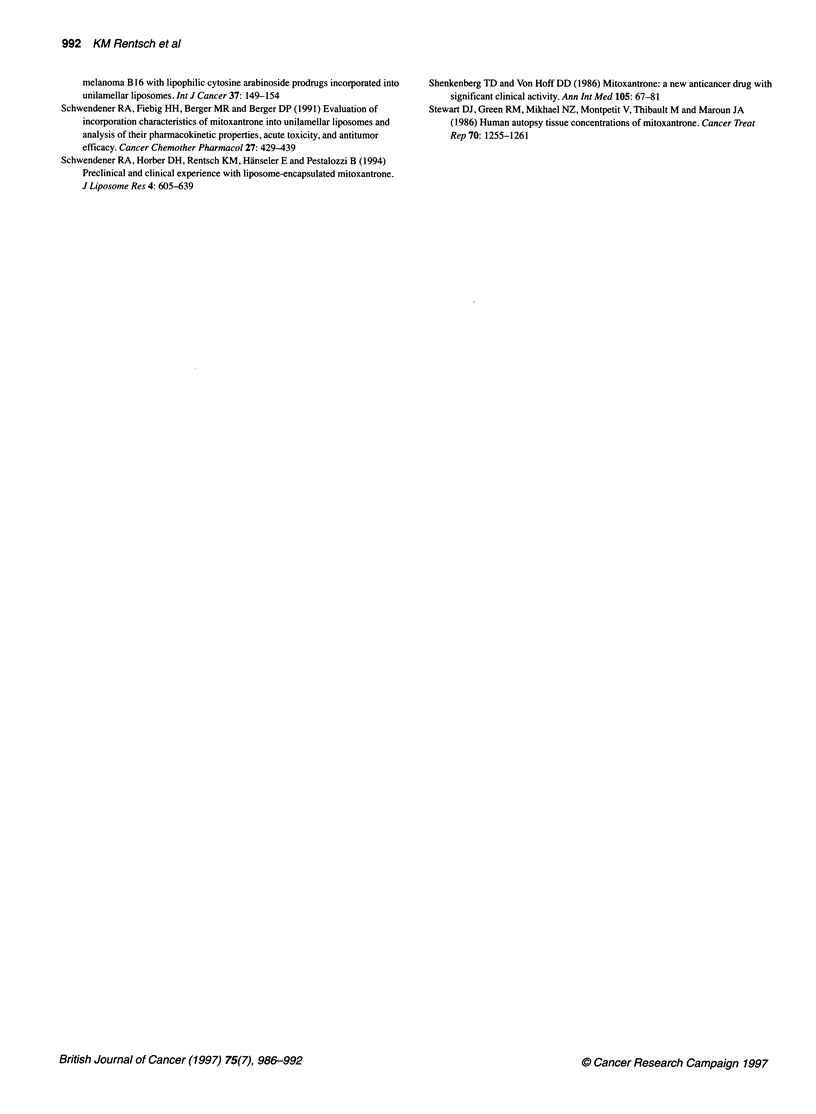# Comparative pharmacokinetic and cytotoxic analysis of three different formulations of mitoxantrone in mice.

**DOI:** 10.1038/bjc.1997.170

**Published:** 1997

**Authors:** K. M. Rentsch, D. H. Horber, R. A. Schwendener, H. Wunderli-Allenspach, E. HÃ¤nseler

**Affiliations:** Institute of Clinical Chemistry, University Hospital ZÃ¼rich, Switzerland.

## Abstract

Two liposomal formulations of mitoxantrone (MTO) were compared with the aqueous solution (free MTO) in terms of their pharmacokinetic behaviour in ICR mice and cytotoxic activity in a nude mouse xenograft model. The three different formulations of MTO [free MTO, phosphatidic acid (PA)-MTO liposomes, pH-MTO liposomes] were administered intravenously (three mice per formulation and time point) at a dose of 4.7 micromol kg(-1) for free MTO, 6.1 micromol kg(-1) for PA-MTO and 4.5 micromol kg(-1) for pH-MTO. The concentrations of MTO were determined using high-performance liquid chromatography (HPLC) in blood, liver, heart, spleen and kidneys of the mice. Additionally, the toxicity and anti-tumour activity of MTO was evaluated in a xenograft model using a human LXFL 529/6 large-cell lung carcinoma. The dose administered was 90% of the maximum tolerated dose (MTD) of the corresponding formulation (8.1 micromol kg(-1) for free MTO, 12.1 micromol kg(-1) for PA-MTO and pH-MTO). The pharmacokinetic behaviour of PA-MTO in blood was faster than that of free MTO, but the cytotoxic effect was improved. In contrast, pH-MTO showed a tenfold increased area under the curve (AUC) in blood compared with free MTO, without improvement of the cytotoxic effect. This discrepancy between the pharmacokinetic and cytotoxic results could be explained by the fact that MTO in pH-MTO liposomes remains mainly in the vascular space, whereas MTO in PA-MTO liposomes is rapidly distributed into deep compartments, even more so than free MTO.


British Journal of Cancer (1997) 75(7), 986–992
© 1997 Cancer Research Campaign

### Comparative pharmacokinetic and cytotoxic analysis of three different formulations of mitoxantrone in mice

**KM Rentsch[1], DH Horber[2], RA Schwendener[2], H Wunderli-Allenspach[3] and E Hänseler[1]**

[1]Institute of Clinical Chemistry, University Hospital Zürich, CH-8091 Zürich; [2]Division of Cancer Research, Department of Pathology, University Hospital Zürich, CH-8091 Zürich; [3]Department of Pharmacy, Swiss Federal Institute of Technology, CH-8057 Zürich, Switzerland

**Summary** Two liposomal formulations of mitoxantrone (MTO) were compared with the aqueous solution (free MTO) in terms of their pharmacokinetic behaviour in ICR mice and cytotoxic activity in a nude mouse xenograft model. The three different formulations of MTO [free MTO, phosphatidic acid (PA)–MTO liposomes, pH–MTO liposomes] were administered intravenously (three mice per formulation and time point) at a dose of 4.7 μmol $kg^{-1}$ for free MTO, 6.1 μmol $kg^{-1}$ for PA–MTO and 4.5 μmol $kg^{-1}$ for pH–MTO. The concentrations of MTO were determined using high-performance liquid chromatography (HPLC) in blood, liver, heart, spleen and kidneys of the mice. Additionally, the toxicity and anti-tumour activity of MTO was evaluated in a xenograft model using a human LXFL 529/6 large-cell lung carcinoma. The dose administered was 90% of the maximum tolerated dose (MTD) of the corresponding formulation (8.1 μmol $kg^{-1}$ for free MTO, 12.1 μmol $kg^{-1}$ for PA–MTO and pH–MTO). The pharmacokinetic behaviour of PA–MTO in blood was faster than that of free MTO, but the cytotoxic effect was improved. In contrast, pH–MTO showed a tenfold increased area under the curve (AUC) in blood compared with free MTO, without improvement of the cytotoxic effect. This discrepancy between the pharmacokinetic and cytotoxic results could be explained by the fact that MTO in pH–MTO liposomes remains mainly in the vascular space, whereas MTO in PA–MTO liposomes is rapidly distributed into deep compartments, even more so than free MTO.

**Keywords:** mitoxantrone; pharmacokinetics; cytotoxicity; mice; liposomes; organ distribution

Mitoxantrone (MTO, Novantrone) or 1,4-dihydroxy-5,8-bis {{2-[(2-hydroxyethyl)-amino]ethyl}amino}-9,10-anthracenedione dihydrochloride is active against lymphomas, breast cancer, acute leukaemias and other malignancies (Shenkenberg and von Hoff, 1986, Faulds et al, 1991). The dose-limiting toxicity of MTO is myelosuppression, but cardiotoxicity may also occur. The risk of cardiomyopathy increases as the total cumulative dose of MTO increases, but it is considerably lower than that with the structurally related anthracyclines. An overall incidence of MTO-associated cardiac effect of 3% in adults and 6% in children has been reported (DuKart et al, 1985); the estimated worst-case incidence of congestive heart failure being 1.3% compared with 2.2% with doxorubicin. For the treatment of solid tumours, MTO is generally administered as a single short-time infusion every 3 weeks at a dose of 12–14 mg $m^{-2}$.

Although MTO features structural similarities to doxorubicin and other DNA-intercalating agents, significant differences in the mechanism of action were found. At least three mechanisms were described: stabilization of the topoisomerase–DNA cleavable complex, which prevents rejoining of strand breaks; aggregation and compaction of DNA via electrostatic cross-linking interactions; and oxidative activation of MTO with free radical generation inducing non-protein-associated strand breaks (Alberts et al, 1985*a*; Blanz et al, 1991; Faulds et al, 1991).

To improve the anti-tumour activity and to reduce toxicity of various other anthracyclines, liposomal formulations were prepared. With doxorubicin (Gabizon et al, 1992; Gabizon, 1993), daunorubicin (Forssen et al, 1992) and epirubicin (Mayhew et al, 1992), it was shown that because of changed pharmacokinetic behaviour (Gabizon et al, 1993) and changed tissue distribution (Forssen et al, 1992) the overall therapeutic index of these anti-tumour drugs could be improved.

Different techniques have been developed to incorporate hydrophilic drugs into liposomes. The first method uses a proton gradient to actively load the liposomes with the drug (Mayer et al, 1986). By lowering the pH in the inner compartment of the liposomes, basic drugs diffuse along the pH gradient into the liposomes, where they interact with the corresponding counterions (e.g. sulphate, citrate) (Mayer et al, 1985; Madden et al, 1991; Gabizon, 1992). The disadvantage of this 'remote loading' technique is the rather low stability of the drug and proton gradient. Another possibility to associate hydrophilic basic drugs with liposomes is to complex them with negatively charged components of the liposome membrane (Amselem et al, 1990; Schwendener et al, 1991). A third technique involves the modification of hydrophilic drugs into lipophilic derivatives (prodrugs). These molecules are incorporated as lipophilic components into the liposomal membrane (Rahman et al, 1986; Rubas et al, 1986).

Two different liposomal formulations were developed with MTO: the first formulation containing phosphatidic acid (PA) to which MTO was complexed (PA–MTO liposomes) (Schwendener et al, 1991) and the second using the 'remote loading' technique (pH–MTO liposomes) (Schwendener et al, 1994). In addition to the different loading techniques of the liposomes with MTO, the pH–MTO liposomes contained poly (ethylene) glycol (PEG)-modified dipalmitoyl phosphatidylethanolamine (PEG(2000)-DPPE) to provide them with long circulating properties (Allen, 1989; Lasic et al, 1991; Gabizon et al, 1993). In order to compare the pharmacokinetic parameters of these two liposomal formulations with the

*Received 24 January 1996*
*Revised 11 October 1996*
*Accepted 16 October 1996*

*Correspondence to:* KM Rentsch, Institute of Clinical Chemistry, University Hospital Zürich, Rämistrasse 100, CH-8091 Zürich, Switzerland aqueous solution that is currently on the market, the concentrations of MTO were measured using high-performance liquid chromatography (HPLC) in blood, liver, heart, spleen and kidneys of mice after intravenous administration of the three formulations. Our goal was to compare the pharmacokinetic properties of MTO in mice after the application of the different pharmaceutical formulations to study the differences in the organ distribution. Additionally, the toxicity and anti-tumour activity of MTO after administration of the different formulations were evaluated in a human xenograft model.

### MATERIALS AND METHODS

### Preparation of MTO formulations

#### Aqueous MTO solution (free MTO)

MTO dihydrochloride [Cyanamid (Schweiz), Lederle Arzneimittel, Adliswil, Switzerland] was dissolved in 0.9% saline. The final concentration was 1261 μmol $l^{-1}$.

#### PA-MTO liposomes (PA-MTO)

Small unilamellar liposomes were prepared by detergent dialysis as described by Schwendener et al (1991). MTO was complexed to PA in the aqueous micellar solution before liposome formation. The acid phosphohydroxy groups of PA associate with the pair of basic secondary amino groups on the side chains of MTO. Because of its amphiphilic properties, the MTO–PA complex is statistically distributed over both membranes of the liposome. The composition of the liposomes was as follows: soy phosphatidylcholine (SPC)/cholesterol/ phosphatidic acid (PA)/MTO/α-tocopherol = 1 : 0.21 : 0.056 : 0.028 : 0.001 (mol). The liposomes were filtered through 0.45-μm sterile filters (Nalge, Rochester, NY, USA) and stored at 4°C. The final concentration of MTO was 766 μmol $l^{-1}$.

#### pH-MTO liposomes (pH-MTO)

MTO was loaded into the aqueous inner compartment of preformed liposomes using a gradient of 10 pH units (Schwendener et al, 1994). Liposomes of the composition SPC/cholesterol/ PEG(2000)-DPPE = 1 : 0.2 : 0.1 (mol) were prepared at pH 2 in ammonium sulphate (0.1 M) by extrusion through 0.1-μm Nucleopore filters. The pH of the external medium was then raised by elution of the liposomes on a Sephadex G75 column which was pre-equilibrated at pH 12. The liposomes were then incubated with 0.2 μM MTO dihydrochloride per 1 μM SPC. Unencapsulated MTO was removed by binding to Dowex 50W×2 (Fluka, Buchs, Switzerland) resin, followed by readjustment of the liposomes to pH 7.4 with phosphate buffer by another column chromatography step. Thioglycerol (1 μl per μmol of MTO) was added as antioxidant, and the liposomes were filtrated with 0.2-μm filters (Acrodisc, Gelman Sciences, Ann Arbor, MI, USA). The final concentration of MTO was 1306 μmol $l^{-1}$.

### Pharmacokinetic analysis in mice

#### Animals

The experiments were performed using female ICR mice (body weight 20–30 g). The animals were housed in air-conditioned rooms on a 12 h/12 h light/dark schedule. Tap water and a commercial, pelleted maintenance diet were fed ad libitum. Either 100 μl of the free MTO and pH-MTO formulations or 200 μl of the PA-MTO liposomes were injected intravenously into the tail vein, corresponding to a dose of 4.7 μmol $kg^{-1}$ for free MTO, 6.1 μmol $kg^{-1}$ for PA-MTO and 4.5 μmol $kg^{-1}$ for pH-MTO. For each pharmaceutical formulation and each time point of measurement, three mice were used. Five and thirty min, 1,2,3 and 24 h after the injection of MTO, the mice were sacrificed by heart puncture under ether anaesthesia, and blood, liver, spleen, heart and kidneys were removed and immediately frozen. To prevent oxidative degradation of mitoxantrone, 20 μl of a solution containing ascorbic acid (100 mg $ml^{-1}$ in 0.1 M citrate buffer pH 3.0) was added to each tube before collecting the blood samples.

#### Sample preparation and drug analysis

The methods for sample preparation and drug analysis of MTO by HPLC have been reported previously (Rentsch et al, 1996). All glassware was silanized using Sylon CT (5% dimethyldichlorosilane in toluene) from Supelco (Bellefonte, PA, USA). Briefly, homogenization of tissues (liver, spleen, heart, kidney) was performed with a potter on ice in a solution of 20% ascorbic acid in 0.1 M citrate buffer (pH 3.0). To 50 mg of tissue, 1 ml of buffer was added. An aliquot of 1 ml of a solution containing hexane sulphonic acid (0.01 mg $ml^{-1}$), ascorbic acid (0.5 mg $ml^{-1}$) and ametantrone (0.08 mg $ml^{-1}$, AMT; Drug Synthesis and Chemistry Branch, Developmental Therapeutics Program, Division of Cancer Treatment, National Cancer Institute, Rockville, MD, USA), as internal standard, was added to 1 ml of tissue homogenate or whole blood. After vortexing for 30 s, 1 ml of 0.1 M borate buffer (pH 9.5) and 300 μl of a 1 N sodium hydroxide solution were added and vortexed again for 30 s. Extraction was performed with 5 ml of dichloromethane on a horizontal shaker (Infors HT, Infors, Bottmingen, Switzerland) during 60 min at 150 r.p.m. After centrifugation for 15 min at 2800 *g*, the organic layer was separated and dried by evaporation (Rotavapor, Büchi, Flawil, Switzerland), and the residue was dissolved in 150 μl of mobile phase.

Samples (92 μl) were injected into a 250 × 4-mm Nucleosil $C_{18}$ column (Macherey Nagel, Oensingen, Switzerland) using an autosampler (9100, Varian, Sunnyvale, CA, USA). The HPLC column was eluted with acetonitrile (33%) and 0.16 M ammonium formate buffer (67%) pH 2.7 at a flow of 1.0 ml $min^{-1}$. Hexane sulphonic acid was added at a concentration of 0.25 M. MTO was quantitated by UV detection using a 9050 UV-VIS Detector (Varian, Sunnyvale, CA, USA) set at 658 nm. The linear range was 4–400 nmol $l^{-1}$ whole blood and 4–1400 nmol $l^{-1}$ tissue homogenate, and the coefficients of variation within-day and between-days were below 4.5% for whole blood and below 10% for tissue homogenates (Rentsch et al, 1996).

#### Pharmacokinetic analysis

All animals were injected i.v. with the same amount of MTO of the respective formulation, irrespective of their body weight. In order to compare directly the pharmacokinetic parameters of the different pharmaceutical formulations, all measured concentrations of MTO were corrected to a dose of 4.5 μmol per kg of body weight and the mean body weight of 27.3 g (standard deviation 2.5 g, $n$ = 54). The concentrations of MTO in the different tissues analysed were corrected for the amount of MTO in the residual tissue blood (Allen, 1989; Khor and Mayersohn, 1991). The area under the curve of drug concentration as a function of time (AUC) and the area under the moment curve (AUMC) were determined with the trapezoidal rule over a period of 24 h for all organs studied. In addition, AUC and AUMC were also determined for infinite time in the case of whole blood.

© Cancer Research Campaign 1997

Calculations were made as follows:

- Mean residence time (MRT):

$$MRT = \frac{AUMC(\infty)}{AUC(\infty)}$$

- Total clearance ($Cl_{tot}$):

$$Cl_{tot} = \frac{Dose}{AUC(\infty)}$$

- Volume of the central compartment ($V_1$):

$$V_1 = \frac{Dose}{c(0)}$$

- Steady-state volume of distribution ($V_{ss}$):

$$V_{ss} = MRT \times Cl_{tot}$$

The initial concentration [$c(0)$] was extrapolated from the intravenous data.

### Toxicity and anti-tumour activity in a human xenograft model

For in vivo experiments, 6- to 8-week-old female athymic nude mice of NMRI genetic background were used. The evaluation of the anti-tumour activity was performed as described earlier (Schwendener et al, 1991). Briefly, tumour slices of human LXFL 529/6 large-cell lung carcinoma were implanted s.c. into both flanks of the animals. The experiment was started after 3–6 weeks when the median tumour diameter had reached 6 mm and the mean body weight of the mice was 28.5 g (standard deviation 3.1 g, $n$ = 12). At day 0, the mice were randomized into treatment groups and control groups each consisting of four mice, which resulted in eight tumours to be evaluated in each group. All compounds were injected as a single dose on day 1 at 90% of the maximal tolerated dose (MTD), which was 8.1 μmol $kg^{-1}$ for free MTO and 12.1 μmol $kg^{-1}$ for PA–MTO and pH–MTO. The control group remained untreated. Tumour growth was recorded weekly by measurement of two perpendicular diameters ($a$,$b$), with ($a \times b^2$) representing the tumour size. The relative tumour volume (RTV) was calculated for each single tumour by dividing the tumour size on the day of evaluation by that on the day of randomization. Median RTV values were used for further evaluation. The anti-tumour effect was evaluated following maximal tumour regression. To estimate toxicity of the different pharmaceutical formulations, the body weight of the mice was monitored during the whole observation period. The relative body weight was calculated by dividing the body weight on the day of evaluation by that on the day of randomization. The death of the animals characterized the end of the observation period.

**Figure 1** The concentration of MTO (μmol $l^{-1}$) (mean and standard deviation) in whole blood as a function of time (min) of free MTO (◆), PA–MTO (■) and pH–MTO (●). The vertical bars represent the standard deviations of the mean ($n$ = 3). The administered doses were 4.7 μmol $kg^{-1}$ MTO with free MTO, 6.1 μmol $kg^{-1}$ with PA–MTO and 4.5 μmol $kg^{-1}$ with pH–MTO

### RESULTS

### Pharmacokinetic analysis in mice

#### *Whole blood*

Mice were injected (i.v. bolus) with each of the three MTO formulations described in Materials and methods. The corresponding concentration–time profiles are shown in Figure 1. Over the whole time of observation, concentrations of MTO were tenfold higher with pH–MTO than with free MTO and PA–MTO. The pharmacokinetic parameters, summarized in Table 1, were calculated from these data with a non-compartmental model. As expected, the area under the curve was highest for pH–MTO, ninefold higher than that for free MTO and 22-fold higher than that for PA–MTO. Accordingly, the mean residence time for pH–MTO was threefold lower than that for free MTO and twofold lower than that for PA–MTO. The total clearance in pH–MTO was ninefold lower than that for free MTO and 20-fold lower than that for PA–MTO. The volume of the central compartment was smallest for pH–MTO, namely sevenfold lower than that for free MTO, and 17-fold lower than that for PA–MTO. Finally, the steady-state volume of distribution for pH–MTO was 29-fold lower than that for free MTO and 37-fold lower than that for PA–MTO.

#### *Tissue distribution*

For all three formulations, the amounts of MTO in whole blood, liver, spleen, heart and kidney were determined as described. Time profiles describing the percentage of the drug in each organ are represented in Figure 2A–C. Comparing identical time points, the amount of MTO being found in a single tissue was consistently higher than that detected in whole blood, with the exception of the 5- and 30-min time points for pH–MTO (Figure 2C). With all formulations, the highest amount of MTO was found in the kidneys. With free MTO and PA–MTO, the maximal concentrations in liver and kidneys were reached within 5 min and in spleen

**Table 1** Pharmacokinetic data of whole blood

| Parameter | Free MTO (mean) | PA–MTO (mean) | pH–MTO (mean) |
|---|---|---|---|
| $c(0)$ (μmol $l^{-1}$) | 9.73 | 3.58 | 64.6 |
| AUC(∞) (μmol min $l^{-1}$) | | | |
| (% extrapolated) | 408 (28.1) | 163 (13.6) | 3516 (5.6) |
| MRT (min) | 1087 | 554 | 325 |
| $Cl_{tot}$ (ml $min^{-1}$) | 0.30 | 0.76 | 0.035 |
| $V_1$ (ml) | 13.0 | 33.1 | 1.99 |
| $V_{ss}$ (ml) | 328 | 419 | 11.4 |

For each formulation and time point, three ICR mice were injected intravenously with 4.7 μmol $kg^{-1}$ free MTO, 6.1 μmol $kg^{-1}$ PA–MTO and 4.5 μmol $kg^{-1}$ pH–MTO. For pharmacokinetic calculations, the concentrations of MTO were corrected to a dose of 4.5 μmol $kg^{-1}$ and a mean body weight of 27.3 g.

© Cancer Research Campaign 1997

**Figure 2** Comparison of the time courses of MTO (% of dose) in heart (●), spleen (▲), whole blood (▽), liver (◆), kidneys (■) and the total recovery (◇) after the application of (**A**) 4.7 μmol $kg^{-1}$ MTO as free MTO, (**B**) 6.1 μmol $kg^{-1}$ MTO as PA–MTO and (**C**) 4.5 μmol $kg^{-1}$ MTO as pH–MTO. The data points represent the percentual values of the means of the amount of MTO in the whole organs determined by HPLC

and heart within 30 min. With pH–MTO, the maximal concentrations in liver, heart and kidneys were reached within 30 min and in spleen within 60 min. For quantitative comparisons, the following parameters were determined: amount of MTO (percent of dose) in the different tissues 5 min after injection, the AUC (24 h), determined per gram organ weight over a period of 24 h and the relative AUC (24 h), i.e. the proportional AUC of a single organ of the total AUC with correction for organ weight (Table 2). The total recovery of MTO 5 min after injection was as follows: for pH–MTO, 107% of the injected dose was recovered with more than 85% in blood; for pH–MTO, a recovery of only 25% of the injected dose was found in the organs that were studied, most of it

**Table 2** Pharmacokinetic data in whole blood and tissues (non-compartmental modelling)[a]

| Parameters | Free MTO (mean) | PA–MTO (mean) | pH–MTO (mean) |
|---|---|---|---|
| *Amount of MTO 5 min after injection (% of dose)*[b] | | | |
| Blood | 11.7 | 4.2 | 88.4 |
| Liver | 25.0 | 14.0 | 10.4 |
| Spleen | 1.1 | 0.3 | 0.6 |
| Heart | 1.6 | 0.3 | 0.5 |
| Kidneys | 35.6 | 6.3 | 7.4 |
| Recovery | 75 | 25 | 107 |
| *AUC (24 h) (μmol min $kg^{-1}$)*[c] | | | |
| Blood | 0.32 | 0.12 | 3.51 |
| Liver | 6.53 | 5.18 | 12.7 |
| Spleen | 19.4 | 5.72 | 24.1 |
| Heart | 12.7 | 4.32 | 7.20 |
| Kidneys | 84.7 | 29.0 | 61.5 |
| *Relative AUC (% of total AUC) (24 h), corrected for organ weight.*[d] | | | |
| Blood | 1.5 | 1.5 | 15.6 |
| Liver | 19.8 | 42.4 | 33.2 |
| Spleen | 4.5 | 3.7 | 5.0 |
| Heart | 3.7 | 2.2 | 1.8 |
| Kidneys | 70.5 | 50.2 | 44.4 |
| Σ | 100 | 100 | 100 |

[a]The dose administered was 4.7 μmol $kg^{-1}$ for free MTO, 6.1 μmol $kg^{-1}$ for PA–MTO and 4.5 μmol $kg^{-1}$ for pH–MTO (three mice per time point and formulation). [b]Amount of MTO, expressed as per cent of dose, found in the different tissues 5 min after injection of MTO. [c]Area under the curve (AUC) determined over a period of 24 h in the different tissues. [d]Relative AUC in the different organs, calculated as per cent of the total AUC in the tissues analysed, which were corrected for organ weight.

in the liver (14%) and less than 5% in the blood; for free MTO, the recovery was 75%, with the highest amount in the kidneys (36%), a considerable amount in the liver (25%) and only 11.7% in the blood. For all three formulations, the amount was low in the spleen (≤ 1.2%). For the quantitative comparison of the various organs, the relative AUC (24 h) with correction for organ weight was used. Again, for all three preparations the highest values were found in the kidneys, followed by the liver. In heart and spleen, values were below 5% in all cases. In whole blood, a value of 16% was found with pH–MTO compared with < 2% for the other two formulations. Comparison between the formulations showed that the relative AUC value in the kidneys was about 50% for the liposomal formulations and 70% for free MTO, whereas in the liver the relative AUC was higher for the liposomal preparations (42% and 33%) compared with free MTO (20%).

### Toxicity and anti-tumour activity in a human xenograft model

### *Toxicity*

During 21 days, the animals of the control group did not change their body weight, whereas all treated animals had a reduced body weight after the chemotherapy. The maximum tolerated dose (MTD) of free MTO in tumour-bearing nude mice was determined to be 9.0 μmol $kg^{-1}$, given i.v. on day 1. After 21 days, the mortality was 20% (one out of five mice) and the median loss of body weight was 8%. For PA–MTO, the MTD was found to be 13.5 μmol $kg^{-1}$, given i.v. on day 1, resulting in a mortality of 0% (none out of four mice) and a median body weight loss of 9% on

© Cancer Research Campaign 1997 

**Figure 3** Median relative tumour volume ($a \times b^2$)/2 (%) of the human xenograft LXFL 529/6 after chemotherapy on day 1 with different pharmaceutical formulations: free MTO (◆), PA–MTO (■), pH–MTO (●) and controls (◇). The administered doses were 8.1 μmol kg$^{-1}$ MTO with free MTO, 12.1 μmol kg$^{-1}$ MTO with PA–MTO and 12.1 μmol kg$^{-1}$ MTO with pH–MTO, respectively, corresponding to 90% of the maximum tolerated dose for the respective formulation day 21. For pH–MTO, the corresponding data were 13.5 μmol kg$^{-1}$ as MTD with a mortality of 25% (one out of four mice) and a median body weight loss of 3%. The minimal relative body weight (RBW) was determined on day 49 for free MTO (RBW 79%), on day 105 for PA–MTO (RBW 67%) and on day 10 for pH–MTO (RBW 83%). The animals treated with free MTO and PA–MTO did not recover during the observation period, whereas the animals treated with pH–MTO recovered after 21 days. Considerable toxicity was found for free MTO at 16 μmol kg$^{-1}$ and at 20 μmol kg$^{-1}$ for PA–MTO and pH–MTO.

### *Efficacy*

The anti-tumour activity of the three formulations was tested in a LXFL 529/6 human xenograft model. The treatment groups received a single i.v. dose of 8.1 μmol kg$^{-1}$ MTO with free MTO and 12.1 μmol kg$^{-1}$ MTO with PA–MTO and pH–MTO. In Figure 3, the relative tumour volume of the LXFL 529/6 human xenograft is shown as a function of time after randomization of the mice. Tumours in control animals grew progressively, showing a median tumour-doubling time of 5 days. With all MTO preparations, partial remissions were achieved (free MTO and pH–MTO within 28 days, PA–MTO within 21 days). The maximal tumour regression was obtained with PA–MTO with a tumour volume of 11% of the initial value, followed by pH–MTO with a tumour volume of 18% of the initial value and free MTO with a tumour volume of 40% of the initial value. To obtain some information on the toxicity of the three different formulations of MTO, changes in weight over time were registered. During 21 days, the control group did not change its body weight, whereas all treated animals had a reduced body weight after the chemotherapy.

### DISCUSSION

Drugs that are liposome bound have firstly to be released from their vehicle to distribute to either plasma proteins, blood cells or different tissues. This complicates the comparison of the pharmacokinetic data in whole blood after the administration of free MTO and the two liposomal formulations (PA–MTO and pH–MTO). Because of analytical difficulties, it was not possible to separate free MTO from the liposome-associated drug. In earlier studies, using [$^{125}$I]tyraminylinulin as liposome marker, 25% of the liposomes could be recovered in blood 10 min after the administration of PA–MTO but only 0.8% of the applied MTO (data not shown). Four hours after the administration of PA–MTO, 25% of the liposomes and only 0.1% of MTO were recovered. With pH–MTO, 60% of the liposomes and 25% of MTO were recovered 10 min after its administration; after 4 h, 40% of the liposomes and 3% of MTO were recovered (Schwendener et al, 1994). These results demonstrate that both the complexation of MTO to PA and the incorporation of MTO using a pH gradient do not generate in vivo a stable liposome formulation with MTO. Therefore, when interpreting the pharmacokinetic results of the three pharmaceutical formulations of MTO, the influence of the liposome-bound amount of MTO can be neglected, at least for the later time points after drug application.

As stated in the Materials and Methods section, we corrected all measured concentrations of MTO to a mean dose of 4.5 μmol kg$^{-1}$ and a mean body weight of 27.3 g for the pharmacokinetic analyses. The highest blood concentrations over the whole time of observation could be determined after the administration of pH–MTO and, accordingly, a significantly larger AUC in blood was calculated than for the other two formulations. Therefore, pH–MTO would be expected to be the preparation with the best cytotoxic activity; the results of the human xenograft model shown in Figure 3 suggest otherwise. The apparent discrepancy can be explained by the pharmacokinetic behaviour of the three formulations. Interesting information was obtained from the comparison of the respective volumes of distribution. For pH–MTO, both the central volume of distribution ($V_1$) and the steady-state volume of distribution ($V_{ss}$) were much smaller than those for the other two formulations. The $V_1$ of pH–MTO (2 ml) corresponded to the total blood volume of mice, suggesting that MTO in this pharmaceutical formulation remained in the central volume immediately after administration. The balance 5 min after the injection of MTO confirmed this result. For pH–MTO, 88% of the administered dose was found in blood 5 min after injection, the respective value for free MTO was 12% and for PA–MTO 4%. At steady state conditions, the volume of distribution of pH–MTO (11 ml) was still lower than the volume of total body water in mice (18 ml). Free MTO and PA–MTO exhibited a much larger $V_{ss}$, indicating that MTO in these pharmaceutical formulations was accumulating in deep compartments. Another pharmacokinetic parameter which allowed interpretation of the divergent results of blood AUC and cytotoxicity was the mean residence time (MRT). It could be demonstrated that although the AUC in blood was much higher with pH–MTO, the MRT of pH–MTO was threefold decreased compared with free MTO. After the administration of pH–MTO, the cytostatic drug was found at high concentrations in blood, but it was not distributed into the different tissues in high quantities. Therefore, the cytotoxic effect on the tumour in the human xenograft model was lower than that observed for free MTO. With PA–MTO, blood levels were about tenfold lower than those determined for pH–MTO but, because of the better tissue penetration of MTO, cytotoxicity was superior. These results support the statement of Liliemark and Peterson (1991) that a higher plasma concentration does not necessarily correlate with a more pronounced cytotoxic effect.

About 18% of the MTO dose administered in its free form is excreted in the faeces within 5 days and approximately 10% was recovered in the urine (Alberts et al, 1985*a*). The fact that MTO is rapidly released from both liposome formulations after i.v. administration implies no changes in the excretion pattern with these pharmaceutical formulations.

© Cancer Research Campaign 1997

In the tissues analysed, 5 min after the administration, the entire amount of MTO was recovered in the case of pH–MTO, three-quarters of the dose were found with free MTO and only one quarter of the dose with PA–MTO (Table 2). This indicates that MTO as free MTO and PA–MTO accumulated in other tissues that were not analysed. In human autopsy tissues, the highest amounts of MTO were found in thyroid, liver, heart, pancreas and spleen in patients with various tumours (Stewart et al, 1986). Alberts et al (1985*b*) reported in their study on the disposition of MTO in cancer patients that MTO appeared to distribute into a deep tissue compartment from which it was slowly released. Roboz et al (1984) stated in a case report that MTO must be distributed in the visceral tissues. Batra et al (1986) reported on the comparative tissue distribution of $^{14}$C-labelled MTO following a single i.v. dose. They demonstrated that MTO accumulates in rats in lung tissue. In contrast, in human studies only small concentrations of MTO are found in this organ. Other tissues with high concentrations of MTO per g of tissue were thyroid and pancreas. After the administration of pH–MTO, MTO remained mainly in blood as we demonstrated by the elevated concentration of MTO in blood over the whole time of observation (Figure 1). The small differences in the amount of MTO administered did not influence the distribution of MTO in the different tissues, as shown by others (Batra et al, 1986).

Cardiotoxicity is the most important side-effect besides the dose-limiting haematological toxicity. After administration of liposomal MTO (either PA–MTO or pH–MTO), the relative AUC in heart tissue was slightly decreased compared with that determined for free MTO. Therefore, a reduced risk of cardiomyopathy can be expected after the administration of the liposomal formulations. The relative AUC in the spleen did not depend on the pharmaceutical formulation, indicating that no accumulation of MTO occurred after injection of the liposomal encapsulated drugs. In contrast, the relative AUC in liver tissue was remarkably increased with PA–MTO compared with free MTO and only slightly increased with pH–MTO. To explain this result, the coating of the liposomes must be considered. Poly(ethylene)glycol-modified dipalmitoyl phosphatidyl-ethanolamine (PEG(2000)-DPPE), the liposome coating used in pH–MTO, is able to significantly inhibit the uptake of the liposomes by the Kupffer cells in the liver (Lasic et al, 1991). In contrast, the PA–MTO liposomes were uncoated and probably carrying negative surface charges and therefore more likely to be rapidly taken up by the mononuclear phagocytic system (MPS).

In conclusion, the pharmacokinetic and cytotoxic behaviour of MTO in mice was compared after administration of three pharmaceutical formulations. pH–MTO showed a tenfold increased AUC in blood compared with free MTO, without improvement of the cytotoxic effect. PA–MTO exhibited faster blood pharmacokinetics than free MTO, but it had an improved cytotoxic effect. This discrepancy between the pharmacokinetic and cytotoxic results could be explained by the fact that MTO in pH–MTO liposomes remained mainly in the vascular space, whereas MTO in PA–MTO liposomes was rapidly distributed into deep compartments, even more so than free MTO.

### ACKNOWLEDGEMENTS

We thank Professor Dr HH Fiebig (University of Freiburg, Germany) for the experiments with the human xenograft model and R Bührer for excellent technical assistance. This study was supported in part by Lederle Arzneimittel GmbH, Wolfratshausen, Germany.

### REFERENCES

Alberts DS, Peng YM, Bowden GT, Dalton WS and Mackel C (1985*a*) Pharmacology of mitoxantrone: mode of action and pharmacokinetics. *Invest New Drugs* **3**: 101–107

Alberts DS, Peng YM, Leigh S, Davis TP and Woodward DL (1985*b*) Disposition of mitoxantrone in cancer patients. *Cancer Res* **45**: 1879–1884

Allen TM (1989) Stealth liposomes: avoiding reticuloendothelial uptake. In *Liposomes in the Therapy of Infectious Diseases and Cancer*, Lopez-Berenstein G and Fidler IJ (eds), pp. 405–415. Alan R Liss: New York

Amselem S, Gabizon A and Barenholz Y (1990) Optimization and upscaling of doxorubicin-containing liposomes for clinical use. *J Pharm Sci* **79**: 1045–1052

Batra VK, Morrison JA, Woodward DL, Siverd NS and Yacobi A (1986) Pharmacokinetics of mitoxantrone in man and laboratory animals. *Drug Metab Rev* **17**: 311–329

Blanz J, Mewes K, Ehninger G, Proksch B, Waidelich D, Greger B and Zeller KP (1991) Evidence for oxidative activation of mitoxantrone in human, pig, and rat. *Drug Metab Dispos* **19**: 871–880

Dukart G, Iatopoulos MJ and Yacobi A (1985) Comment on mitoxantrone. *Drug Intell Clin Pharm* **19**: 216–218

Faulds D, Balfour JA, Chrisp P and Langtry HD (1991) Mitoxantrone. A review of its pharmacodynamic and pharmacokinetic properties, and therapeutic potential in the chemotherapy of cancer.*Drugs* **41**: 400–449

Forssen EA, Coulter DM and Proffitt RT (1992) Selective in vivo localization of daunorubicin small unilamellar vesicles in solid tumors. *Cancer Res* **52**: 3255–3261

Gabizon AA (1992) Selective tumor localization and improved therapeutic index of anthracyclines encapsulated in long-circulating liposomes. *Cancer Res* **52**: 891–896

Gabizon AA, Barenholz Y and Bialer M (1993) Prolongation of the circulation time of doxorubicin encapsulated in liposomes containing a polyethylene glycol-derivatized phospholipid: pharmacokinetic studies in rodents and dogs. *Pharm Res* **10**: 703–708

Khor SP and Mayersohn M (1991) Potential error in the measurement of tissue to blood distribution coefficients in physiological pharmacokinetic modelling. Residual tissue blood. I. Theoretical considerations. *Drug Metab Disp* **19**: 478–485

Lasic D, Martin FJ, Gabizon A, Huang SK and Papahadjopoulos D (1991) Sterically stabilized liposomes: a hypothesis on the molecular origin of the extended circulation time. *Biochim Biophys Acta* **1070**: 187–192

Liliemark J and Peterson C (1991) Pharmacokinetic optimisation of anticancer therapy. *Clin Pharmacokin* **21**: 213–231

Madden LD, Harrigan PR, Tai LCL, Bally MB Mayer LD, Redelmeier TE, Loughrey HC, Tilcock CPS, Reinish LW and Cullis PR (1990) The accumulation of drugs within large unilamellar vesicles exhibiting a proton gradient: a survey. *Chem Phys Lipids* **53**: 37–46

Mayer LD, Bally MB, Hope MJ and Cullis PR (1985) Uptake of antineoplastic agents into large unilamellar vesicles in response to a membrane potential. *Biochim Biophys Acta* **816**: 294–302

Mayer LD, Bally MB and Cullis PR (1986) Uptake of adriamycin into large unilamellar vesicles in response to a pH gradient. *Biochim Biophys Acta* **857**: 123–126

Mayhew EG, Lasic D, Babbar S and Martin FJ (1992) Pharmacokinetics and antitumor activity of epirubicin encapsulated in long-circulationg liposomes incorporating a polyethylene glycol-derivatized phospholipid. *Int J Cancer* **51**: 302–309

Rahman A, Ganjei A and Neefe JR (1986) Comparative immunotoxicity of free doxorubicin and doxorubicin encapsulated in cardiolipin liposomes. *Cancer Chemother Pharmacol* **16**: 28–34

Rentsch KM, Schwendener RA and Hänseler E (1996) Determination of mitoxantrone in mouse whole blood and different tissues by high performance liquid chromatography. *J Chromat Biomed B* **679**: 185–192

Roboz JP, Paciucci A, Silides D, Greaves J and Holland JF (1984) Detection and quantification of mitoxantrone in human organs. *Cancer Chemother Pharmacol* **13**: 67–68

Rubas W, Supersaxo A, Weder HG, Hartmann HR, Hengartner H, Schott H and Schwendener RA (1986) Treatment of murine L1210 lymphoid leukemia and melanoma B16 with lipophilic cytosine arabinoside prodrugs incorporated into unilamellar liposomes. *Int J Cancer* **37**: 149–154

Schwendener RA, Fiebig HH, Berger MR and Berger DP (1991) Evaluation of incorporation characteristics of mitoxantrone into unilamellar liposomes and analysis of their pharmacokinetic properties, acute toxicity, and antitumor efficacy. *Cancer Chemother Pharmacol* **27**: 429–439

Schwendener RA, Horber DH, Rentsch KM, Hänseler E and Pestalozzi B (1994) Preclinical and clinical experience with liposome-encapsulated mitoxantrone. *J Liposome Res* **4**: 605–639

Shenkenberg TD and Von Hoff DD (1986) Mitoxantrone: a new anticancer drug with significant clinical activity. *Ann Int Med* **105**: 67–81

Stewart DJ, Green RM, Mikhael NZ, Montpetit V, Thibault M and Maroun JA (1986) Human autopsy tissue concentrations of mitoxantrone. *Cancer Treat Rep* **70**: 1255–1261

© Cancer Research Campaign 1997